# Model of single-sized endotracheal tube for adults

**DOI:** 10.31744/einstein_journal/2020AO4805

**Published:** 2019-10-10

**Authors:** Luiz Guilherme Calderon, Marcos Mello Moreira, Gilson Barreto, Alfio José Tincani

**Affiliations:** 1 Faculdade de Ciências Médicas, Universidade Estadual de Campinas, Campinas, SP, Brazil.

**Keywords:** Airway resistance, Intubation, intratracheal, Tracheal intubation, Flow rate

## Abstract

**Objective:**

To simulate different diameters of endotracheal tubes and to verify the fluid dynamics aspects by means of flow and resistance measurements.

**Methods:**

Fluid dynamics software was used to calculate mean flow and airway resistance in endotracheal tube with a diameter of 6.0, 7.0, 7.5, 8.0, 9.0 and 10.0mm at normal body temperature and under constant pressure. The same measurements were taken in the fusion of the first 22cm of a 9.0mm endotracheal tube with 10.0mm diameter, and with the end part in 12cm of a 6.0mm endotracheal tube with 7.0mm diameter.

**Results:**

The fusion of the first 22cm of an endotracheal tube of 10.0mm diameter with the terminal part in 12cm of an endotracheal tube of 6.0mm diameter, preserving the total length of 34cm, generated average flow and airway resistance similar to that of a conventional 7.5mm endotracheal tube.

**Conclusion:**

This simulation study demonstrates that a single-sized endotracheal tube may facilitate endotracheal intubation without causing increased airway resistance.

## INTRODUCTION

Orotracheal intubation (OTI) is a procedure used in cases requiring invasive mechanical ventilation, and was first described by Andreas Vesalius, in 1543.^1 - 4^ An endotracheal tube (ETT) not only enables efficient ventilation, but also prevents the possible entry of gastric and oral contents into the lungs, when fitted with a cuff.^3^

The idea of a modified ETT first appeared in the literature in 1945, when Cole^5 , 6^ proposed a model to be used in neonatal and pediatric patients. This model gained popularity since it proposed easy introduction of the device, and decrease by approximately 50% of flow resistance as compared to conventional cylindrical ETT, with direct impact on reduced respiratory work.^7^

A single ETT for adults should, above all, preserve the ideal ventilatory conditions for optimal mechanical ventilation, avoiding increased airway resistance (AWR). This characteristic could be based on Poiseuille’s law, which states that the flow (F) through a given tube results from the difference in pressure (P) from one end to the other, in tube length (l) and radius (r), and fluid viscosity (μ).^8^ It is applied to rigid tubes with constant radius, and expressed by the following formula:

$$\frac{F\;=\;µr^{4}P}{8µL}$$

The flow is directly proportional to the radius to the fourth power, therefore the tube diameter represents the most important factor determining the flow velocity of a fluid passing through it.^9^

## OBJECTIVE

To verify the aspects of fluid dynamics considering flow and resistance measurements in different diameters of conventional and fused endotracheal tubes.

## METHODS

The Computational Fluid Dynamics (CFD) software was used in the *Laboratório Nacional de Luz Síncrotron* , in the city of Campinas (State of São Paulo, Brazil), in the Engineering Division in order to calculate the mean flow in ETT of different diameters.

**Figure 1:**
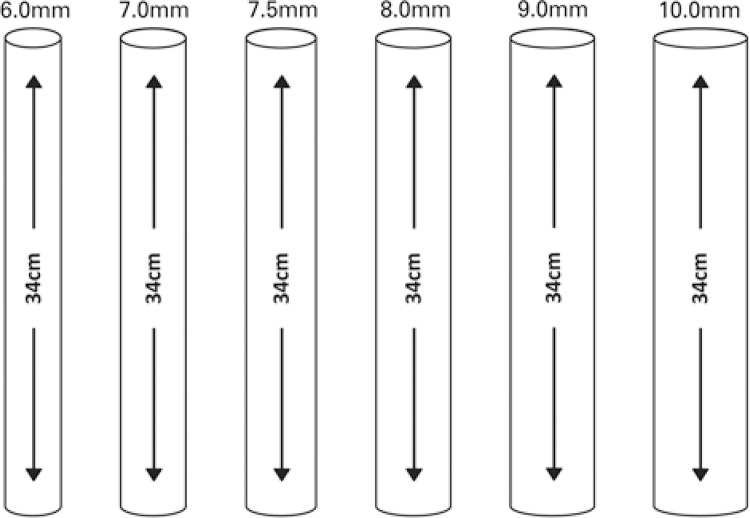
Six models of conventional endotracheal tube used, with their diameter and length

**Figure 2:**
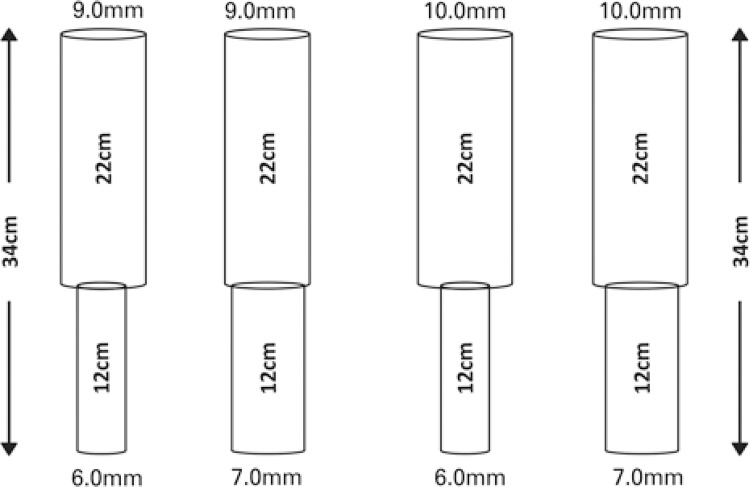
Four models of fused endotracheal tubes used, showing the length for each diameter and the total length

The simulations were conducted using a constant 25cmH_2_O pressure, at body temperature of 36°C in ETT with total length of 34cm. The mean flow and calculated airway resistance values (cAWR) were recorded in two data points:

- Data point 1 − conventional ETT (cETT): the flow velocity at the tube entrance and exit, and cAWR were measured in cETT with diameters of 6.0, 7.0, 7.5, 8.0, 9.0, and 10.0cm ( Figure 1 ).

- Data point 2 – Fused ETT (fETT): using the CFD, we developed four types of ETT, which were called fETT. The tubes were fused with 22 plus 12 cm, totaling up 34cm of cETT. The 9.0mm and 10.0mm tubes were 22cm long, and the 6.0mm and 7.0mm tubes were 12cm long, generating the fETT: 9.0/6.0mm; 10.0/6.0mm; 9.0/7.0mm; 10.0/7.0mm ( Figure 2 ).

The flow velocities at the entrance and exit, and cAWR in fETT were measured. In both data points, the value of the entrance and exit flow velocities for each ETT was added and divided by two in order to obtain the mean flow.

### Ethical aspects

This is a simulation study with the measurements of fluid dynamic of an ETT model calculated by a software. This research was not conducted with human beings and/or animals, therefore it was exempt of submission to the Internal Review Board (Resolution CNS 466/12).

## RESULTS

For the mean flow and cAWR results, the ANSYS software was used; they are depicted on tables 1 and 2 . In a 10.0/6.0mm fETT, the mean flow was 99.6Lpm and the cAWR was 15.69cmH_2_O/L/s – similar to those obtained with the 7.5mm cETT.

**Table 1:** Calculated airway flow and resistance in conventional endotracheal tubes of different diameters

Diameter (mm)	Mean flow (Lpm)	cAWR (cmH_2_O/L/s)
6.0	64.3	23.34
7.0	92.6	16.19
7.5	105.7	14.20
8.0	127.0	11.81
9.0	168.0	8.92
10.0	209.3	7.16

cAWR: calculated airway resistance.

**Table 2:** Calculated airway flow and resistance in fused endotracheal tubes of different diameters

Diameter (mm)	Mean flow (Lpm)	cAWR (cmH_2_O/L/s)
9.0/6.0	94.8	15.82
10.0/6.0	99.6	15.69
9.0/7.0	127.7	11.74
10.0/7.0	134.6	11.14

cAWR: calculated airway resistance.

## DISCUSSION

Invasive mechanical ventilation is often necessary for successful treatment of acute respiratory failure. It is, therefore, considered as an important measure capable of saving the lives of critically ill patients.^10^

The development of a single-size ETT for the adult population, with variable diameter along the length, seems to be an alternative that does not increase airflow resistance, facilitates OTI especially in the emergency setting, and reduces the need for a wide repertoire of device sizes available to physicians.

Cole tracheal tube was developed by the anesthesiologist Frank Cole in 1945,^5^ when endotracheal anesthesia in infants and young children was still rare. Endotracheal anesthesia refers to the administration of anesthetic gases through an ETT inserted through the oropharynx or nasopharynx, until it reaches the larynx and trachea. Infant ETT must have a narrow diameter to access the larynx. The narrower tube diameters cause greater airflow resistance and can increase respiratory work. Cole created his ETT to be narrower only where it should be positioned: below the larynx.^11^ Considering Cole’s hypothesis for adult patients, tubes with smaller diameter would also cause greater resistance during ventilation.

All prototype ETT designed to date focus on the pediatric population and are still rarely used.^12^ If developed for adult patients, such models could generate benefits as greater chance of success when inserting an ETT. In addition, a possible reduction in expenditure could be considered.

The results found in this study demonstrated that the 8.0mm cETT presented mean flow (127.0Lpm) and cAWR (11.81cmH_2_O/L/s) similar to a 9.0/7.0 fETT (mean flow (127.7Lpm), and cAWR (11.74cmH_2_O/L/s). However, despite the similar values, the use of the 9.0/7.0mm fETT might not be as favorable as the 10.0/6.0mm fETT as to the ease of intubating the patient with a smaller distal diameter tube.

The idea of using a fETT with a distal diameter of 6.0mm is to facilitate intubation in the urgency and emergency setting, and it can also be used in elective intubations for surgical procedures. Further studies are needed to determine the need for cuff, or whether this prototype could be developed tapered, in order to eliminate the cuff. However, in this simulation study, cuff ETTs were not developed, and further studies are required to assess the need of cuffs.

### Study limitations

Since it is a software-based study, some variables, such as body temperature and adjusted pressure, may generate variations at the entrance and exit flows measured, as compared to a bench study.

## CONCLUSION

Fluid dynamics using a software suggests that the fusion of the initial 22cm of a 10.0mm endotracheal tube to the final 12cm of a 6.0mm endotracheal tube can be correlated with a conventional 7.5mm endotracheal tube regarding the mean flow and calculated airway resistance.

The elimination of conventional endotracheal tube sizes 6.0mm, 6.5mm, 7.0mm, and 7.5mm can lower the costs of medical services. Since this analysis was not included in the objectives of this study, further studies are necessary to determine this estimate.

Having a single-sized endotracheal tube may be advantageous, for facilitating orotracheal intubation with a fused 10.0/6.0mm endotracheal tube without causing increased airway resistance. However, this is only a computational simulation study.
