# Supplementary material for: Model of single-sized endotracheal tube for adults
Source: Einstein (Sao Paulo). 2019 Oct 10;18:eAO4805. doi: 10.31744/einstein_journal/2020AO4805 (PMC6896656; doi:10.31744/einstein_journal/2020AO4805)
Supplement: Translation

# Translation: Modelo de tubo endotraqueal de tamanho único para adultos

**Authors:** Luiz Guilherme Calderon, Marcos Mello Moreira, Gilson Barreto, Alfio José Tincani

**Affiliations:** 1 Faculdade de Ciências Médicas, Universidade Estadual de Campinas, Campinas, SP, Brasil.

**Keywords:** Resistência das vias respiratórias, Intubação intratraqueal, Intubação traqueal, Taxa de fluxo

## Abstract

**Objetivo:**

Simular diferentes diâmetros de tubos endotraqueais e verificar os aspectos fluidinâmicos, considerando medições de fluxo e resistência.

**Métodos:**

Foi utilizado um software de fluidinâmica para calcular o fluxo médio e a resistência das vias aéreas nos tubos endotraqueais com diâmetro de 6,0, 7,0, 7,5, 8,0, 9,0 e 10,0mm, em temperatura corporal normal e pressão constante. As mesmas medidas foram realizadas na fusão dos primeiros 22cm de um tubo endotraqueal de 9,0 e 10,0mm de diâmetro, com a parte terminal em 12cm de um tubo endotraqueal de 6,0 e 7,0mm de diâmetro.

**Resultados:**

A fusão dos primeiros 22cm de um tubo endotraqueal de diâmetro 10,0 mm com a parte terminal em 12cm de um tubo endotraqueal de 6,0mm de diâmetro, preservando o comprimento total de 34cm, gerou fluxo médio e resistência de vias aéreas semelhantes aos de um tubo endotraqueal convencional de 7,5mm.

**Conclusão:**

Um tubo endotraqueal de tamanho único pode facilitar a intubação endotraqueal, sem causar aumento de resistência na via aérea.

## INTRODUÇÃO

A intubação orotraqueal (IOT), procedimento utilizado em casos em que há necessidade de ventilação mecânica invasiva, foi descrita pela primeira vez em 1543 por Andreas Vesalius.^1 - 4^ Um tubo endotraqueal (TET) permite não somente uma ventilação eficiente, mas também evita a possível entrada de conteúdo gástrico e oral para o interior dos pulmões, quando dotado de balonete ou *cuff* .^3^

A ideia de um TET modificado já data na literatura desde 1945, quando Cole^5 , 6^ propôs um modelo para ser utilizado em neonatologia e pediatria. Esse modelo ganhou popularidade devido à proposta de fácil introdução do dispositivo e redução de aproximadamente 50% da resistência ao fluxo em relação ao TET cilíndrico convencional, com impacto direto na diminuição do trabalho respiratório.^7^

Um TET único para adultos deve, sobretudo, preservar a condição ventilatória ideal para a ventilação mecânica ótima, evitando aumento da resistência das vias aéreas (Rva). Essa característica pode ser baseada na lei de Poiseuille, que estabelece que o fluxo (F), por meio de um determinado tubo, é resultado da diferença de pressão (P) de uma extremidade a outra, do comprimento (l) e raio (r) desse tubo, além da viscosidade (µ) do fluido.^8^ É aplicada a tubos rígidos, raios constantes e expressa pela seguinte fórmula:

$$\frac{F\;=\;µr^{4}P}{8µL}$$

O fluxo é diretamente proporcional à quarta potência do raio, o que demonstra que o diâmetro do tubo representa o papel principal dentre todos os fatores determinativos da velocidade do fluxo de certo fluido por meio deste.^9^

## OBJETIVO

Verificar os aspectos fluidinâmicos considerando medições de fluxo e resistência em diferentes diâmetros de tubos endotraqueais convencionais e fundidos.

## MÉTODOS

Foi utilizado o software *Computational Fluid Dynamics* (CFD), no Laboratório Nacional de Luz Síncrotron em Campinas (SP) Brasil, na Divisão de Engenharia para calcular o fluxo médio em diferentes diâmetros de TET.

As simulações foram realizadas utilizando pressão constante de 25cmH_2_O, temperatura corporal de 36°C em TETs com comprimento total de 34cm. Os valores de fluxo médio e resistência calculada de vias aéreas (Rcva) foram registrados em dois momentos:

- Momento 1 − TET convencional (TETc): foram medidos os valores da velocidade de entrada e saída de fluxo e Rcva em TETc com diâmetros de 6,0, 7,0, 7,5, 8,0, 9,0 e 10,0cm ( Figura 1 ).

**Figura 1:** Seis modelos de tubo endotraqueal convencional utilizados, com seu diâmetro e comprimento

- Momento 2 − TET fundido (TETf): desenvolvemos, pelo CFD, quatro tipos de TET, que denominamos de TETf. Os tubos foram fundidos com 22 mais 12cm, totalizando o tamanho de 34cm do TETc. Os diâmetros de 9,0 e 10,0mm tinham 22cm, e os diâmetros de 6,0 e 7,0mm tinham 12cm de comprimento, criando os TETf: 9,0/6,0mm; 10,0/6,0mm; 9,0/7,0mm; 10,0/7,0mm ( Figura 2 ).

**Figura 2:** Quatro modelos de tubos endotraqueais fundidos utilizados, demonstrando o comprimento em cada diâmetro e comprimento total

Foram medidos os valores da velocidade de entrada e saída de fluxo e Rcva em TETf. Em ambos os momentos, o valor do fluxo de entrada e saída de cada TET foi somado e dividido por dois, para obter o fluxo médio.

### Aspectos éticos

Trata-se de estudo de simulação *,* envolvendo medidas de fluidodinâmica de um modelo de TET, calculadas por um software. Por não se tratar de pesquisa envolvendo seres humanos e/ou animais, o presente estudo dispensou submissão à apreciação da Comissão de Ética em Pesquisa (Resolução CNS 466/12).

## RESULTADOS

Os resultados de fluxo médio e Rcva foram obtidos utilizando o software da ANSYS e estão descritos na tabela 1 e 2 . Em um TETf de 10,0/6,0mm, o fluxo médio foi de 99,6Lpm e a Rcva de 15,69cmH_2_O/L/s − valores semelhantes aos de um TETc de 7,5mm.

**Tabela 1:** Cálculo de fluxo e resistência calculada de vias aéreas em diferentes diâmetros de tubo endotraqueal convencional

Diâmetro (mm)	Fluxo médio (Lpm)	Rcva (cmH_2_O/L/s)
6,0	64,3	23,34
7,0	92,6	16,19
7,5	105,7	14,20
8,0	127,0	11,81
9,0	168,0	8,92
10,0	209,3	7,16

Rcva: resistência calculada de vias aéreas.

**Tabela 2:** Cálculo de fluxo e resistência calculada de vias aéreas em diferentes diâmetros de tubo endotraqueal fundido

Diâmetro (mm)	Fluxo médio (Lpm)	Rcva (cmH_2_O/L/s)
9,0/6,0	94,8	15,82
10,0/6,0	99,6	15,69
9,0/7,0	127,7	11,74
10,0/7,0	134,6	11,14

Rcva: resistência calculada de vias aéreas.

## DISCUSSÃO

A ventilação mecânica invasiva é frequentemente necessária para o sucesso do tratamento da insuficiência respiratória aguda. Assim, considera-se como medida importante e capaz de salvar a vida dos pacientes em estado crítico.^10^

O desenvolvimento de um TET de tamanho único para a população adulta, com diâmetro variável ao longo do comprimento, parece ser alternativa que não aumenta a resistência ao fluxo de ar, facilita a IOT especialmente em cenários de urgência e emergência, e reduz a necessidade de amplo repertório de tamanhos de dispositivos à disposição do médico.

O tubo traqueal de Cole foi desenvolvido pelo anestesiologista Frank Cole, em 1945,^5^ quando a anestesia endotraqueal em lactentes e crianças pequenas ainda era incomum. Anestesia endotraqueal refere-se à administração de gases anestésicos por meio de um TET inserido através da orofaringe ou nasofaringe, até alcançar a laringe e traqueia. Os TET infantis precisam ter diâmetro estreito para acessar a laringe. Os diâmetros dos tubos mais estreitos causam mais resistência ao fluxo de ar através do tubo e podem aumentar o trabalho respiratório. Cole criou seu TET para ser mais estreito apenas onde deveria estar posicionado: abaixo da laringe.^11^ Considerando a mesma hipótese de Cole, para pacientes adultos, tubos com menor diâmetro também causam maior resistência durante a ventilação.

Todos os protótipos dos TETs projetados até o momento se concentram na população pediátrica e ainda são raramente utilizados.^12^ Se desenvolvidos para os pacientes adultos, tais modelos poderiam gerar benefícios como maior chance de sucesso ao inserir o TET. Além disso, uma possível redução nas despesas poderia ser avaliada.

Os resultados encontrados neste estudo demonstraram que o TETc de 8,0mm apresentou fluxo médio (127,0Lpm) e Rcva (11,81cmH_2_O/L/s) semelhantes a um TETf 9,0/7,0 em relação ao fluxo médio (127,7Lpm) e Rcva (11,74cmH_2_O/L/s). No entanto, apesar dos valores serem próximos, a utilização do TETf 9,0/7,0mm poderia não ser tão benéfica em comparação ao TETf 10,0/6,0mm em relação à facilidade de intubar o paciente com um tubo que possui diâmetro distal menor.

A ideia de utilizar um TETf com diâmetro distal de 6,0mm é facilitar a intubação em cenários de urgência e emergência, e que também possa ser utilizado nas intubações eletivas, para procedimentos cirúrgicos. Além disso, ainda são necessários mais estudos para determinar a necessidade de balonete, ou se esse protótipo poderia ser desenvolvido em formato cônico, podendo eliminar o *cuff.* Porém, neste estudo de simulação, não foram desenvolvidos TET com *cuff* , e outros estudos são necessários para avaliar a necessidade ou não do balonete.

### Limitações do estudo

Por se tratar de um estudo utilizando um software computacional, algumas variáveis, como temperatura corporal e pressão ajustada, podem gerar variações nos valores medidos de fluxo de entrada e saída, quando comparados a um estudo de bancada.

## CONCLUSÃO

A fluidodinâmica utilizando um software sugere que a fusão de 22cm iniciais de um tubo endotraqueal com 10,0mm de diâmetro e os 12cm terminais de um tubo endotraqueal com 6,0mm de diâmetro pode correlacionar-se com tubo endotraqueal convencional de 7,5mm em relação ao fluxo médio e resistência calculada de vias aéreas.

A eliminação dos tamanhos de tubo endotraqueal convencional de diâmetros 6,0mm, 6,5mm, 7,0mm e 7,5mm pode gerar redução nos custos de serviços médicos. Como essa análise não foi parte integrante dos objetivos deste trabalho, estudos futuros são necessários para determinar essa estimativa.

Ter um tubo endotraqueal de tamanho único pode ser benéfico, já que poderia facilitar a intubação orotraqueal com a utilização de um tubo endotraqueal fundido de 10,0/6,0mm, sem causar aumento da resistência calculada de vias aéreas. No entanto, este é apenas um estudo de simulação computacional.
